# Single-step multiplex reverse transcription-polymerase chain reaction for the detection and differentiation of QX-like infectious bronchitis virus from the Thai variant and vaccine strains H120, Ma5, and 4/91

**DOI:** 10.14202/vetworld.2023.1109-1113

**Published:** 2023-05-27

**Authors:** Sucheeva Junnu, Tawatchai Pohuang

**Affiliations:** 1 Division of Livestock Medicine, Faculty of Veterinary Medicine, Khon Kaen University, Khon Kaen 40002, Thailand; 2 Research Group for Emerging and Re-emerging Infectious Diseases in Animals and Zoonotic Diseases, Faculty of Veterinary Medicine, Khon Kaen University, Khon Kaen 40002, Thailand

**Keywords:** detection, QX-like infectious bronchitis virus, single-step multiplex reverse transcription-polymerase chain reaction, specificity

## Abstract

**Background and Aim:**

QX-like infectious bronchitis virus (IBV) is a highly infectious avian coronavirus that causes respiratory and kidney disease. It is linked to increased mortality and loss of performance in infected chickens worldwide, including Thailand. Thus, a simple and rapid diagnostic method for the diagnosis of QX-like IBV is needed. This study aimed to develop a single-step multiplex reverse transcription-polymerase chain reaction (mRT–PCR) assay to detect and differentiate QX-like IBV from Thai IBV and vaccine strains used in the poultry industry (H120, Ma5, and 4/91).

**Materials and Methods:**

Primer sets specific for QX-like and Thai IBV were designed to target the S1 gene. The specificity of the technique was verified using nine isolates of QX-like IBV, four isolates of Thai IBV, and other avian viral respiratory pathogens. The detection limit was evaluated using a serial ten-fold dilution of QX-like and Thai IBV.

**Results:**

The results showed that single-step mRT–PCR could detect QX-like IBV and differentiate it from Thai IBV and the vaccine strains H120, Ma5, and 4/91. The limit of detection of the developed assay was 10^2.2^ embryo infectious dose (EID)_50_/mL for QX-like IBV and 10^1.8^ EID_50_/mL for Thai IBV. Interestingly, the developed assay could identify mixed infection by both IBVs in a single sample.

**Conclusion:**

The single-step mRT–PCR assay developed in this study can potentially discriminate QX-like IBV from Thai IBV and the vaccine strains H120, Ma5, and 4/91 in a single reaction. It is also suitable for use in all laboratories with access to conventional PCR equipment.

## Introduction

Infectious bronchitis (IB) caused by IB virus (IBV) is considered endemic in poultry-producing regions worldwide. Infectious bronchitis causes significant economic losses in the poultry industry due to increased mortality in infected birds. Infectious bronchitis virus belongs to the genus *Gammacoronavirus* and family *Coronaviridae* [1]. In recent years, the Thai poultry industry has experienced several outbreaks of IB caused by QX-like IBV [2, 3], which has also been detected in several countries in the past decade [4]. The QX genotype (currently renamed as GI-19 lineage) [5], which was described for the 1^st^ time in 1996 in China [6], has spread from Asia to other regions and become the most prevalent field strain in many countries such as Italy, the Netherlands, France, United Kingdom, Ukraine, Hungary and Turkey [4, 7]. Infectious bronchitis clinically manifests as respiratory distress, nephritis, decreased egg production, and poor egg quality [8]. In addition to QX-like IBV, Thailand has experienced outbreaks of Thai variant IBV. Previously, Thai variant IBV, also known as group I Thai IBV, was reported to circulate in chicken farms in some regions of Thailand. Interestingly, this recombinant virus originated from parental strains belonging to the Thai local strain and QX-like IBV [3]. Although Thai IBV is a recombinant virus, it was clustered into the GI-19 lineage based on the Valastro classification [2].

Vaccination is routinely used in commercial chicken farms to prevent economic losses from IBV infection. However, there is a low level or no cross-protection against IBV strains [9, 10]. Because the vaccination of flocks does not always prevent IB outbreaks, a rapid and simple diagnostic method for detecting the causative virus and differentiating field strains from vaccine strains is highly desirable. Conventional laboratory diagnosis of QX-like IBV is based on virus isolation in embryonated chicken eggs, followed by virus detection and confirmation through reverse transcription-polymerase chain reaction (RT–PCR) and sequencing of the RT–PCR product [4, 7, 8]. However, this method is laborious and time-consuming. In recent years, progress in the field of molecular biology has facilitated the development of rapid and reliable diagnostic assays. Real-time RT–PCR for IBV detection has been developed, which provides highly specific and sensitive results in a timely manner [11–13]. However, real-time RT-PCR requires more specialized devices. Thus, rapid and sensitive diagnostic techniques for verifying the clinical diagnosis of QX-like IBV are needed. Reverse transcription-polymerase chain reaction is an alternative method for IBV detection and has a similar ability to detect IBV as virus isolation in embryonated chicken eggs [14]. Multiplex RT-PCR (mRT–PCR)-based methods have the advantages of being simple and rapid, with no requirements for specialized laboratory equipment other than conventional PCR instruments. Multiplex RT-PCR for IBV typing has been reported, but only a limited number of IBV strains have been differentiated [15, 16].

This study aimed to develop a single-step mRT–PCR assay to detect and differentiate QX-like IBV from Thai IBV and vaccine strains used in the poultry industry (H120, Ma5, and 4/91).

## Materials and Methods

### Ethical approval

Ethical approval is not needed to pursue this type of study.

### Study period and location

This study was conducted from January 2021 to June 2022 at the Faculty of Veterinary Medicine, Khon Kaen University, Khon Kaen, Thailand.

### Viruses

Nine isolates of QX-like IBV and four isolates of Thai IBV used in this study were confirmed via RT–PCR and S1 gene sequencing as described previously [3]. They were stored in allantoic fluid at −80°C until use. The IBV vaccine strains H120 Bioral H120 (Boehringer Ingelheim, Ingelheim, Germany), Ma5 (Nobilis^®^ IB Ma5, MSD Animal Health, New Jersey, USA), and 4/91 (Nobilis^®^ IB 4/91) were included in this study.

### Primers

Primer sets specific for QX-like and Thai IBV were designed to target the S1 gene. Primers F55N2-TGA TTG ATA CTG CCA GTA AT and R436-ACA ATG TGT GAC AAA CAG TG, amplifying a 271-bp product, were used to detect QX-like IBV. Primers F173-CGC AGG TGG TTC CTC TGA AT and R436-ACA ATG TGT GAC AAA CAG TG, amplifying a 158-bp product, were used to detect Thai IBV. The primer sets used to amplify all IBV strains included GU391-GCT TTT GAG CCT AGC GTT, according to a previous report [3], and GL494-ACC AGA ACC TGT CAC CTC, which was selected from a highly conserved region of 5′-UTR. These primers amplified a 103-bp product for use in the developed assay. Basic local alignment search tool (BLAST) analysis of the GenBank database deposited in National Center for Biotechnology Information (NCBI) (http://www.ncbi.nlm.nih.gov/BLAST/) was performed to confirm the specificity of the primers.

### Multiplex reverse transcription-polymerase chain reaction protocol

Viral RNA was extracted from 200 μL of allantoic fluid using Viral Nucleic Acid Extraction Kit (Real Biotech, Taiwan) following the manufacturer’s protocol. The resulting RNA was dissolved in 50 μL of nuclease-free water. Single-step RT–PCR (AccessQuick™ RT-PCR System, Promega, USA) was performed according to the manufacturer’s instructions. The reverse transcription was performed at 48°C for 45 min followed by 94°C for 5 min. The PCR program consisted of 35 cycles of 94°C for 30 s, 56°C for 30 s, and 72°C for 30 s, followed by a 10-min extension at 72°C. Reverse transcription-polymerase chain reaction products were analyzed on a 2% agarose gel containing RedSafe Nucleic Acid Staining Solution (JH Science, Kirkland, WA, USA). The gels were run at 120 V for 45 min and visualized using a Gel Doc™ XR+ Gel Documentation System (Bio-Rad, Hercules, CA, USA).

### Comparative limit of detection

To determine the limit of detection of the developed single-step mRT–PCR assay, it was performed using ten-fold serial dilutions of the QX-like IBV isolate THA80151 ranging from 10^6.2^ to 10^0.2^ embryo infectious dose (EID)_50_/mL and the Thai IBV isolate THA90151 ranging from 10^6.8^ to 10^0.8^ EID_50_/mL. Each dilution of the virus was also tested after isolation from embryonated chicken eggs. The limit of detection of virus isolation was set as the first dilution at which no embryo mortality or lesions were observed.

### Specificity test

The specificity of the assay was tested using nine isolates of QX-like IBV; four isolates of Thai IBV; and the vaccine strains H120, Ma5, and 4/91. Other avian viral respiratory pathogens were also tested, including Newcastle disease virus (NDV) and infectious laryngotracheitis virus (ILTV).

### Identification of mixed IBV

To evaluate the ability of the developed assay to detect mixed IBV, RNA from THA80151 and THA90151 was mixed at equal volumes and tested using the developed single-step mRT–PCR assay.

## Results

### Infectious bronchitis virus detection and typing

**Figure-1:**
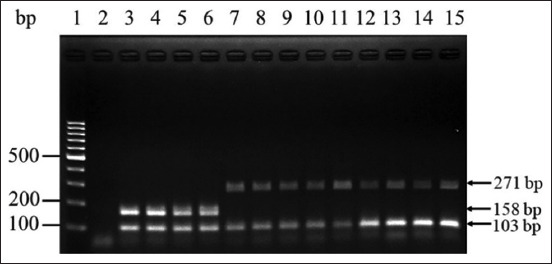
The single-step multiplex reverse transcription-polymerase chain reaction of Thai variant infectious bronchitis virus (IBV) and QX-like IBV. Lane 1: 100 bp DNA ladder; Lane 2: negative control; Lanes 3–6: four isolates of Thai variant IBV; and QX-like lane 7–15: nine isolates of QX-like IBV.

**Figure-2:**
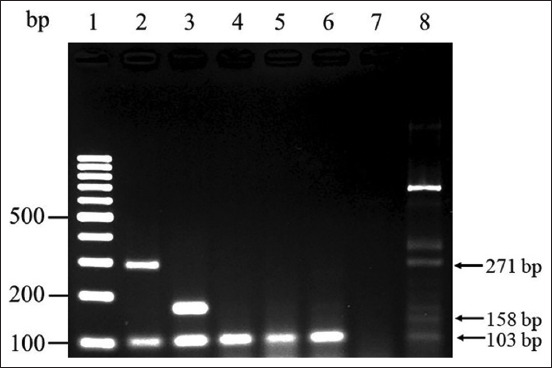
Specificity of the single-step multiplex reverse transcription-polymerase chain reaction. Lane 1: 100 bp DNA ladder; Lane 2: QX-like infectious bronchitis virus (IBV) (represented by THA80151); Lane 3: Thai variant IBV (represented by THA90151); Lanes 4–6: vaccine strain H120, Ma5 and 4/91, respectively; lane 7: Infectious laryngotracheitis virus; and lane 8: Newcastle disease virus.

**Figure-3:**
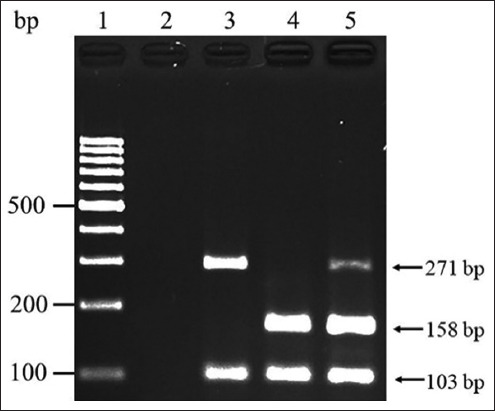
The single-step multiplex reverse transcription-polymerase chain reaction for mixed RNA sample of QX-like and Thai variant infectious bronchitis virus (IBV). Lane 1: 100 bp DNA ladder; Lane 2 negative control; Lane 3: QX-like IBV (represented by THA80151); Lane 4: Thai variant IBV (represented by THA90151); and Lane 5: Mixed sample.

As shown in Figures-1 and 2, the examined IBVs, namely, QX-like IBV, Thai IBV, H120, Ma5, and 4/91, were differentiated based on the corresponding bands on agarose gels after single-step mRT–PCR. QX-like and Thai IBV were distinguished by specific bands at 271 and 158 bp, respectively. However, neither of these bands was observed after amplifying the sequences of the vaccine strains H120, Ma5, and 4/91 (Figure-2). Furthermore, this single-step mRT–PCR assay could detect both QX-like and Thai IBV in a mixed RNA sample (Figure-3). In addition, the expected 103-bp product of all IBV strains used in this study could be observed after single-step mRT–PCR amplification (Figures-1 and-2).

### Detection limits and specificity

**Figure-4:**
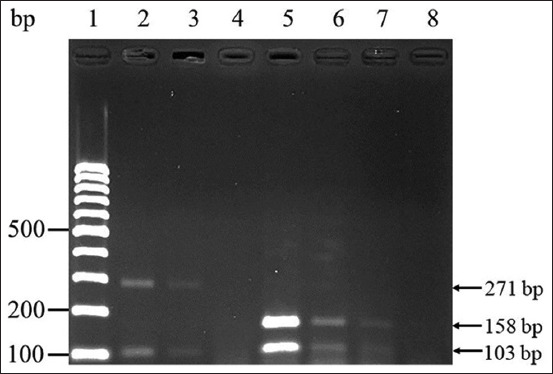
Detection limits of single-step multiplex reverse transcription-polymerase chain reaction. Lane 1: 100 bp DNA ladder; Lanes 2–4: serial ten-fold dilutions of QX-like infectious bronchitis virus (IBV) (represented by THA80151) ranging from 10^3.2^ to 10^1.2^ EID_50_/mL.; Lanes 5–8: serial ten-fold dilutions of Thai variant IBV (represented by THA90151) ranging from 10^3.8^ to 10^0.8^ EID_50_/mL. EID=Embryo infectious dose.

QX-like and Thai IBV were detected and discriminated based on the expected bands. No bands were detected when ILTV was tested in this single-step mRT–PCR assay. Unexpected bands were generated after NDV amplification, but they did not correspond to the bands obtained for IBV (Figure-2). The limits of detection of the single-step mRT–PCR assay were 10^2.2^ EID_50_/mL (10^−4^ dilution) for QX-like IBV and 10^1.8^ EID_50_/mL (10^−5^ dilution) for Thai IBV (Figure-4). Further, virus isolation revealed the limits of detection of 10^1.2^ EID_50_/mL (10^−5^ dilution) for QX-like IBV and 10^0.8^ EID_50_/mL (10^−6^ dilution) for Thai IBV.

## Discussion

Currently, QX-like IBV is the predominant genotype of IBV in Thailand [2]. The spread of QX-like IBV is controlled through the use of live attenuated vaccines, which is difficult because different serotypes of vaccines do not provide adequate protection [17, 18]. Therefore, rapid detection of the virus within an infected chicken flock is important for controlling QX-like IBV outbreaks. In addition, chicken flocks can be appropriately vaccinated against this highly infectious virus. We developed a single-step mRT–PCR assay in the present study to detect and differentiate QX-like IBV from Thai IBV and the vaccine strains H120, Ma5, and 4/91.

The single-step mRT–PCR assay developed in this study could simultaneously detect and differentiate QX-like IBV from Thai IBV and the vaccine strains H120, Ma5, and 4/91 in a single reaction. Furthermore, the developed assay could detect mixed IBV in a single sample, as specific RT–PCR products were obtained for the two viruses. The specificity of the developed assay was also tested using other viral respiratory pathogens. No RT–PCR product was generated for ILTV (species *Gallid alphaherpesvirus* 1), and the RT–PCR product generated for NDV was inconsistent with that generated for IBV. These findings indicated that the developed single-step mRT–PCR assay was specific for IBV. The limits of detection of the developed assay were 10^2.2^ EID_50_/mL for QX-like IBV and 10^1.8^ EID_50_/mL for Thai IBV. Although the limits of detection of the developed assay were higher than those of virus isolation, they were similar to the detection limits of real-time mRT–PCR assay for the classical (G1) and Egyptian variant II (G23) IBV strains. According to a previous study [12], real-time mRT-PCR could detect viral RNA at a concentration of 10^2^ EID_50_/mL for both genotypes. Our study suggested that this single-step mRT–PCR was a rapid and sensitive assay for discriminating QX-like and Thai IBV. Moreover, it was a useful method for the diagnosis of QX-like and Thai IBV infection in chicken flocks vaccinated with H120, Ma5, and 4/91.

In molecular diagnostic assays, the S1 gene is widely used in the design of strain-specific primers for IBV detection [11, 15, 16] because it is associated with strain heterogeneity and biological characteristics [19]. This study selected the specific primers for QX-like and Thai IBV from S1 gene sequences. The results showed that the single-step mRT–PCR assay could generate RT–PCR products with the expected sizes for the individual strains tested in this study. Because infections by different strains of IBV other than QX-like and Thai IBV can occur in the field, the single-step mRT–PCR assay should also have the ability to detect general IBV. Therefore, we added a pair of primers targeting the 5′-UTR of the IBV genome in our assay that may amplify all IBV strains. We revealed that the primers could detect all tested IBV strains, consistent with a report by Callison *et al*. [20]. The highly conserved 5′-UTR of the IBV genome has been used as a target region for the real-time TaqMan^®^-based RT–PCR assay, which could amplify all IBV strains [20]. This suggested that our single-step mRT–PCR assay has the potential to detect general IBV.

## Conclusion

A single-step mRT–PCR assay was developed for the rapid and accurate typing of QX-like IBV and its discrimination from Thai IBV and vaccine strains used in the poultry industry (H120, Ma5, and 4/91). In addition, the method discriminated QX-like IBV from other avian viral respiratory pathogens. Hence, the single-step mRT–PCR assay developed in this study can be used as a diagnostic assay for detecting QX-like IBV, and it is suitable for use in all laboratories with access to conventional PCR equipment.

## Authors’ Contributions

TP: Conceptualization, supervision, and project administration. SJ and TP: Methodology, data analysis, writing, reviewing, and editing. All authors have read, reviewed, and approved the final manuscript.
